# Assessment of differentially expressed plasma microRNAs in nonsyndromic cleft palate and nonsyndromic cleft lip with cleft palate

**DOI:** 10.18632/oncotarget.13379

**Published:** 2016-11-16

**Authors:** Jingyun Li, Jijun Zou, Qian Li, Ling Chen, Yanli Gao, Hui Yan, Bei Zhou, Jun Li

**Affiliations:** ^1^ State Key Laboratory of Reproductive Medicine, Department of Plastic and Cosmetic Surgery, Maternal and Child Health Medical Institute, Obstetrics and Gynecology Hospital Affiliated to Nanjing Medical University, Nanjing 210004, China; ^2^ Department of Burns and Plastic Surgery, Children's Hospital of Nanjing Medical University, Nanjing 210008, China

**Keywords:** nonsyndromic cleft palate, nonsyndromic cleft lip with cleft palate, plasma microRNA, miRNA microarray

## Abstract

Plasma microRNAs (miRNAs) have recently emerged as a new class of regulatory molecules that influence many biological functions. However, the expression profile of plasma microRNAs in nonsyndromic cleft palate (NSCP) or nonsyndromic cleft lip with cleft palate (NSCLP) remains poorly investigated. In this study, we used Agilent human miRNA microarray chips to monitor miRNA levels in three NSCP plasma samples (mixed as the CP group), three NSCLP plasma samples (mixed as the CLP group) and three normal plasma samples (mixed as the Control group). Six selected plasma miRNAs were validated in samples from an additional 16 CP, 33 CLP and 8 healthy children using qRT-PCR. Using Venn diagrams, distinct and overlapping dysregulated miRNAs were identified. Their respective target genes were further assessed using gene ontology and pathway analysis. The results show that distinct or overlapping biological processes and signalling pathways were involved in CP and CLP. Our study showed that the common key gene targets reflected functional relationships to the Notch, Wnt, phosphatidylinositol and Hedgehog signalling pathways. Further studies should examine the mechanism of the potential target genes, which may provide new avenues for future clinical prevention and therapy.

## INTRODUCTION

Orofacial clefts include cleft palate only (CPO), cleft lip with cleft palate (CLP) and cleft lip only (CLO). Approximately 1/800 live births worldwide are affected by these diseases [1]. Nonsyndromic orofacial clefts occur as isolated entities with no other apparent structural and/or developmental abnormalities. The majority of CLP cases are nonsyndromic (NS) (∼70%) [2]. Cleft palate only (CPO) is the least common form of the orofacial clefts (approximately 33%) [3]. The aetiology is multifactorial and involves both genetic and environmental risk factors. Most studies suggest that distinct etiological mechanisms underlie CLP and CPO [4, 5]; however, some overlapping exists in their aetiologies [6, 7]. Thus, our knowledge about whether CPO does indeed differ from CLP remains incomplete.

Mounting evidence suggests that miRNAs could be functionally important for the regulation of vertebrate and mammalian orofacial clefting [8–10]. The identification of the miRNA-mRNA regulatory molecules is important to understand the regulatory mechanisms of CLP and CPO. The potential of plasma miRNAs to be potential non-invasive diagnostic biomarkers for nonsyndromic cleft lip in infants has been reported [11]. In this study, based on our Agilent human miRNA microarray chips, we identified differentially expressed plasma microRNAs in nonsyndromic cleft palate and nonsyndromic cleft lip with cleft palate. Using gene ontology and pathway analysis of the target genes of these miRNAs, we report that distinct biological processes interact and coordinate the physiology of nonsyndromic cleft palate and nonsyndromic cleft lip with cleft palate.

## RESULTS

### miRNA microarray analysis

**Figure 1:**
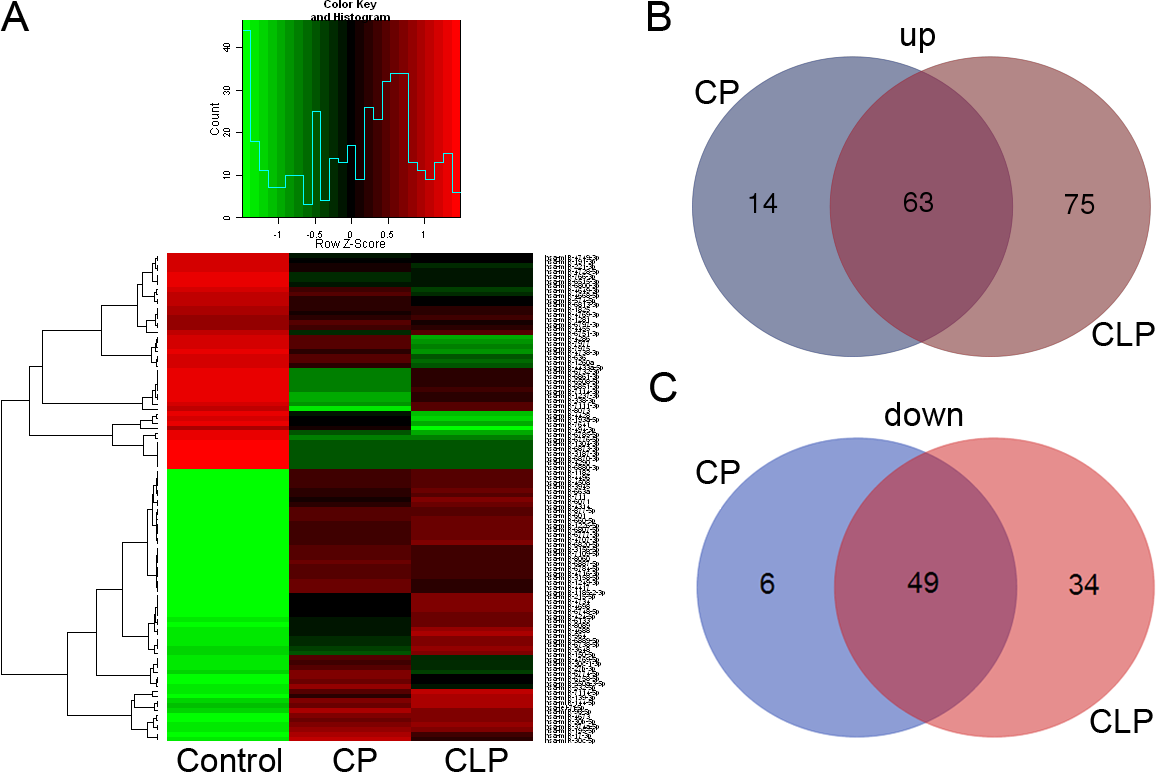
plasma miRNA microarray expression data among the patients with nonsyndromic cleft palate, nonsyndromic cleft lip with cleft palate and healthy children (**A**) Hierarchical clustering reveals the miRNA expression profile. (**B**) Venn diagrams show the number of distinct and overlapping upregulated miRNAs in CP and CLP. (**C**) Venn diagrams show the number of distinct and overlapping downregulated miRNAs in CP and CLP.

To investigate whether circulating miRNAs are associated with the pathogenesis of cleft palate and cleft lip with cleft palate, plasma samples were collected from healthy children and children with nonsyndromic cleft palate (NSCLP) and cleft lip with cleft palate (NSCLP). A comprehensive miRNA microarray analysis was performed on nine plasma samples, including three NSCP plasma samples (mixed as the CP group), three NSCLP plasma samples (mixed as the CLP group) and three plasma samples from healthy children (mixed as the Control group). Hierarchical clustering was used to show the miRNAs expression variation and patterns among these three groups CP, CLP and Control (Figure 1A). We also screened for differentially expressed miRNAs that showed a two-fold or greater change (compared to the control group) in the CP and CLP plasma samples, and illustrated the overlapping between the two data sets using Venn diagrams (Figure 1B, 1C). A total of 63 miRNAs were upregulated in both the CP and CLP plasma samples (Figure 1B). The upregulated miRNAs are listed in Table 1 (fold change ≥ 2). Furthermore, 49 miRNAs were downregulated in both the CP and CLP plasma samples (Figure 1C). The downregulated miRNAs are listed in Table 2 (fold change ≥ 2).

**Table 1:** List of 63 miRNAs that were co-overexpressed in CP and CLP plasma samples

miRNA	Fold change in CP	Fold change in CLP	Mirbase_accession_No
hsa-let-7a-5p	2.593625	3.62266	MIMAT0000062
hsa-let-7f-5p	36.22597	94.70817	MIMAT0000067
hsa-miR-1182	21.12385	26.03614	MIMAT0005827
hsa-miR-1185-2-3p	35.17025	22.02562	MIMAT0022713
hsa-miR-1226-5p	33.54898	41.68948	MIMAT0005576
hsa-miR-1249-3p	40.65461	26.77842	MIMAT0005901
hsa-miR-126-5p	22.59582	11.5428	MIMAT0000444
hsa-miR-139-3p	9.764356	30.04384	MIMAT0004552
hsa-miR-144-5p	22.1287	34.8168	MIMAT0004600
hsa-miR-150-5p	2.004063	5.130724	MIMAT0000451
hsa-miR-17-3p	25.74652	10.66161	MIMAT0000071
hsa-miR-194-5p	2.004321	2.671813	MIMAT0000460
hsa-miR-195-5p	11.29176	10.05747	MIMAT0000461
hsa-miR-215-5p	10.32331	24.41001	MIMAT0000272
hsa-miR-27b-3p	10.79146	4.974262	MIMAT0000419
hsa-miR-301a-3p	2.216769	2.039103	MIMAT0000688
hsa-miR-30b-5p	43.5225	53.10868	MIMAT0000420
hsa-miR-30c-1-3p	9.047681	4.742653	MIMAT0004674
hsa-miR-30c-5p	10.41076	4.763296	MIMAT0000244
hsa-miR-3156-5p	39.43275	33.64425	MIMAT0015030
hsa-miR-3158-5p	36.59335	29.4613	MIMAT0019211
hsa-miR-340-5p	4.435901	10.44386	MIMAT0004692
hsa-miR-3648	2.844126	7.529149	MIMAT0018068
hsa-miR-374a-5p	41.436	53.56016	MIMAT0000727
hsa-miR-3945	54.65085	55.2666	MIMAT0018361
hsa-miR-424-5p	11.5428	23.91295	MIMAT0001341
hsa-miR-4314	48.6155	91.10153	MIMAT0016868
hsa-miR-4417	39.91649	27.81709	MIMAT0018929
hsa-miR-4496	21.48931	25.86879	MIMAT0019031
hsa-miR-4508	20.71299	22.6453	MIMAT0019045
hsa-miR-4673	23.30205	21.36562	MIMAT0019755
hsa-miR-4688	9.853577	37.87732	MIMAT0019777
hsa-miR-4698	2.111651	2.899994	MIMAT0019793
hsa-miR-4707-3p	53.56016	88.45938	MIMAT0019808
hsa-miR-4716-3p	25.26925	22.1287	MIMAT0019827
hsa-miR-4734	10.01005	24.63449	MIMAT0019859
hsa-miR-4769-5p	10.66161	4.959765	MIMAT0019922
hsa-miR-532-5p	10.62257	4.541014	MIMAT0002888
hsa-miR-550a-3-5p	22.36834	10.8752	MIMAT0020925
hsa-miR-564	9.835221	47.83535	MIMAT0003228
hsa-miR-601	35.01135	36.03937	MIMAT0003269
hsa-miR-6071	21.2377	52.38286	MIMAT0023696
hsa-miR-6133	10.44386	27.50138	MIMAT0024617
hsa-miR-652-3p	22.6453	4.860917	MIMAT0003322
hsa-miR-660-5p	24.63449	28.21952	MIMAT0003338
hsa-miR-663a	28.76448	47.35553	MIMAT0003326
hsa-miR-6738-5p	9.491392	40.3395	MIMAT0027377
hsa-miR-6748-5p	10.37443	20.60795	MIMAT0027396
hsa-miR-6758-5p	23.39996	9.81465	MIMAT0027416
hsa-miR-6774-5p	20.60795	4.699301	MIMAT0027448
hsa-miR-6777-3p	35.46738	53.8186	MIMAT0027455
hsa-miR-6784-5p	33.23554	28.4822	MIMAT0027468
hsa-miR-6793-5p	23.91295	3.695676	MIMAT0027486
hsa-miR-6807-5p	27.50138	34.4576	MIMAT0027514
hsa-miR-6820-5p	22.91788	39.11779	MIMAT0027540
hsa-miR-6887-5p	28.4822	22.30914	MIMAT0027674
hsa-miR-6889-5p	9.57147	39.91649	MIMAT0027678
hsa-miR-7109-5p	22.30914	20.89674	MIMAT0028115
hsa-miR-711	21.61314	28.39723	MIMAT0012734
hsa-miR-7114-5p	9.41187	23.30205	MIMAT0028125
hsa-miR-8060	29.71088	24.85582	MIMAT0030987
hsa-miR-8089	9.716781	22.36834	MIMAT0031016
hsa-miR-877-5p	36.42551	38.19915	MIMAT0004949

**Table 2:** List of 49 miRNAs that were co-downregulated in CP and CLP plasma samples

miRNA	Fold change in CP	Fold change in CLP	Mirbase_accession_No
hsa-miR-122-5p	0.014823713	0.330486	MIMAT0000421
hsa-miR-1237-3p	0.04279959	0.178651	MIMAT0005592
hsa-miR-1260a	0.42197306	0.188688	MIMAT0005911
hsa-miR-1281	0.292045994	0.361803	MIMAT0005939
hsa-miR-1304-3p	0.022515879	0.021418	MIMAT0022720
hsa-miR-1825	0.305341608	0.315741	MIMAT0006765
hsa-miR-191-3p	0.210027916	0.202306	MIMAT0001618
hsa-miR-193a-5p	0.425119659	0.1725	MIMAT0004614
hsa-miR-221-3p	0.38537867	0.317683	MIMAT0000278
hsa-miR-3187-3p	0.013885943	0.013209	MIMAT0015069
hsa-miR-338-3p	0.04279959	0.214453	MIMAT0000763
hsa-miR-4286	0.481616926	0.148742	MIMAT0016916
hsa-miR-4290	0.04279959	0.040714	MIMAT0016921
hsa-miR-4428	0.354170872	0.170319	MIMAT0018943
hsa-miR-4433a-5p	0.207866953	0.084119	MIMAT0020956
hsa-miR-4455	0.422613454	0.365739	MIMAT0018977
hsa-miR-4649-3p	0.200187171	0.091757	MIMAT0019712
hsa-miR-4668-5p	0.389545483	0.170031	MIMAT0019745
hsa-miR-4728-5p	0.471992677	0.412945	MIMAT0019849
hsa-miR-4738-3p	0.356057291	0.151948	MIMAT0019867
hsa-miR-4749-3p	0.431131255	0.458677	MIMAT0019886
hsa-miR-4769-3p	0.299500434	0.328468	MIMAT0019923
hsa-miR-483-3p	0.025346248	0.25173	MIMAT0002173
hsa-miR-494-3p	0.421072365	0.074374	MIMAT0002816
hsa-miR-497-5p	0.26762079	0.401373	MIMAT0002820
hsa-miR-574-3p	0.032438225	0.165143	MIMAT0003239
hsa-miR-574-5p	0.398577728	0.302998	MIMAT0004795
hsa-miR-636	0.405198518	0.173279	MIMAT0003306
hsa-miR-6508-5p	0.021489753	0.091996	MIMAT0025472
hsa-miR-6515-3p	0.233030948	0.255185	MIMAT0025487
hsa-miR-6732-3p	0.029496564	0.129832	MIMAT0027366
hsa-miR-6751-3p	0.096354218	0.272251	MIMAT0027403
hsa-miR-6776-5p	0.023690732	0.022536	MIMAT0027452
hsa-miR-6785-5p	0.292436258	0.215104	MIMAT0027470
hsa-miR-6797-3p	0.486474133	0.356753	MIMAT0027495
hsa-miR-6800-3p	0.207096926	0.209525	MIMAT0027501
hsa-miR-6813-3p	0.295100077	0.244702	MIMAT0027527
hsa-miR-6851-3p	0.02840522	0.109846	MIMAT0027603
hsa-miR-6861-3p	0.02773473	0.119237	MIMAT0027624
hsa-miR-6870-3p	0.04279959	0.040714	MIMAT0027641
hsa-miR-6873-3p	0.022068915	0.020993	MIMAT0027647
hsa-miR-6880-3p	0.04279959	0.040714	MIMAT0027661
hsa-miR-7111-3p	0.02234751	0.227218	MIMAT0028120
hsa-miR-7114-3p	0.04279959	0.180041	MIMAT0028126
hsa-miR-7641	0.466679177	0.29544	MIMAT0029782
hsa-miR-766-3p	0.248388555	0.280315	MIMAT0003888
hsa-miR-7975	0.443053554	0.153591	MIMAT0031178
hsa-miR-7977	0.425407891	0.15303	MIMAT0031180
hsa-miR-8073	0.04279959	0.462327	MIMAT0031000

### Expression validation of selected miRNAs using Bulge-Loop™ qRT-PCR analysis

**Figure 2:**
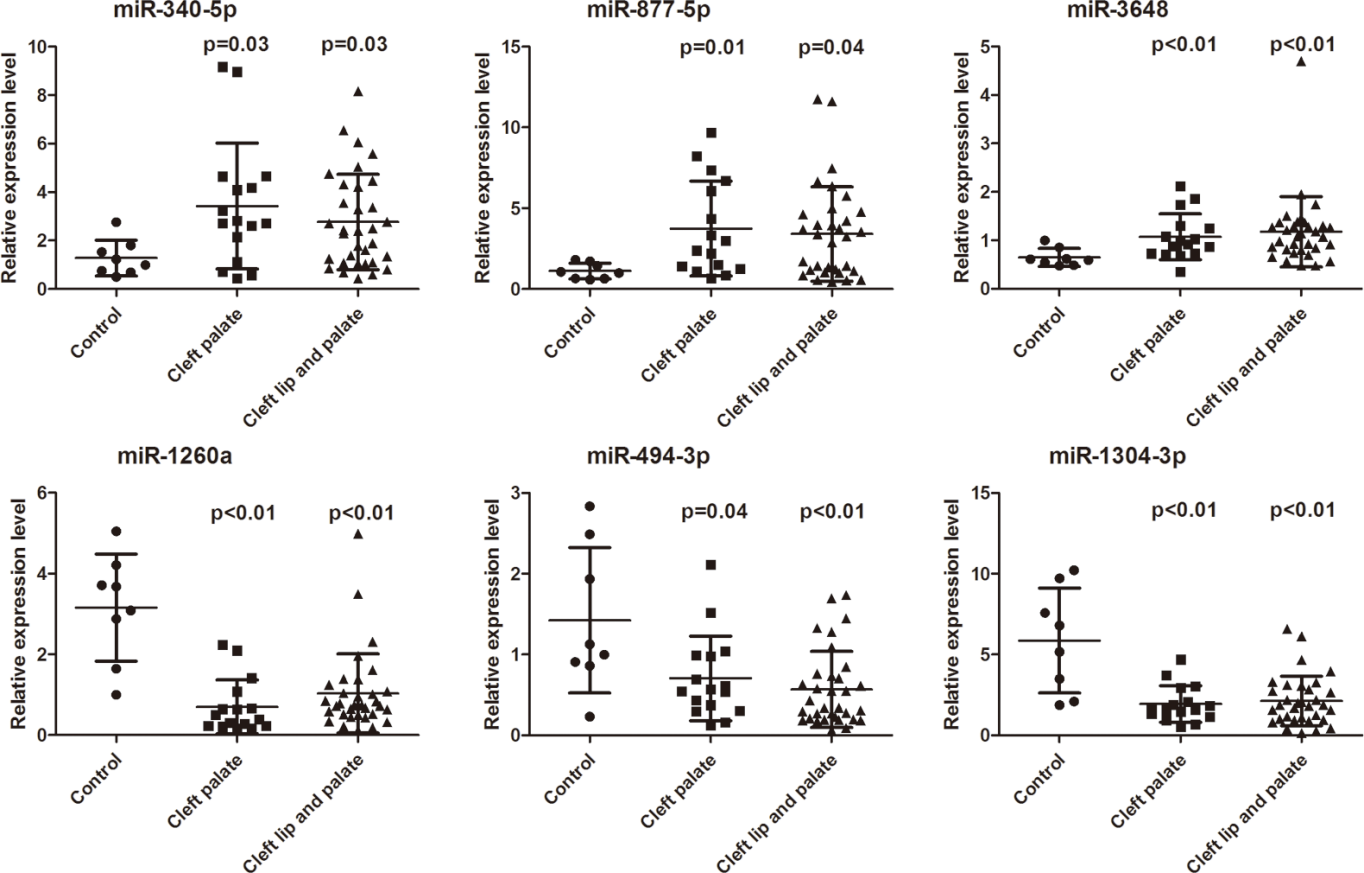
Relative expression level (2^−ΔΔCt^) of miR-340-5p, miR-877-5p, miR-3648, miR-1260a, miR-494-3p, and miR-1304-3p expression in the plasma of cleft palate patients (*n* =16), cleft lip with cleft palate patients (*n* = 33) and controls (*n* = 8) (Mann-Whitney *U* test) *P* values are listed above the data dot.

Among the 63 upregulated and 49 downregulated miRNAs in both the CP and CLP plasma samples, six miRNAs, namely miR-340–5p, miR-877–5p, miR-3648, miR-1260a, miR-494–3p, and miR-1304–3p, were selected for expression validation (Table 3). The selection standards were the same as we previously reported [11] and were as follows: infrequently reported in the literature, present in both samples and higher deep sequencing reads in miRBase. Bulge-Loop™ qRT-PCR was performed to validate these six differentially expressed miRNAs found in the miRNA microarray analysis. RNA was isolated from 57 plasma samples, including samples from 16 CP, 33 CLP and 8 healthy children, using the mirVana PARIS kit (Ambion, Carlsbad, CA, USA). The results showed that three miRNAs, miR-340–5p, miR-877–5p and miR-3648, were significantly upregulated in both the CP and CLP plasma samples (Figure 2). In contrast, three miRNAs, miR-1260a, miR-494–3p and miR-1304–3p, were significantly downregulated in both the CP and CLP plasma samples (Figure 2). Therefore, a similar pattern of upregulation and downregulation was observed in both the microarray and qRT-PCR samples for the 6 miRNAs assessed (Table 3, Figure 2). Therefore, our microarray data were reliable and stable.

**Table 3:** Selected six miRNAs’ basic characteristics

miRNA	Fold change in CP	Fold change in CLP	Dysregulation	miRBase deep sequencing reads
miR-340-5p	4.435901	10.44386	up	329
miR-877-5p	36.42551	38.19915	up	2840
miR-3648	2.844126	7.529149	up	650
miR-1260a	0.42197306	0.188688	down	29
miR-494-3p	0.421072365	0.074374	down	153
miR-1304-3p	0.022515879	0.021418	down	4

### Functional analysis of potential target genes of co-expressed dysregulated miRNAs

**Figure 3:**
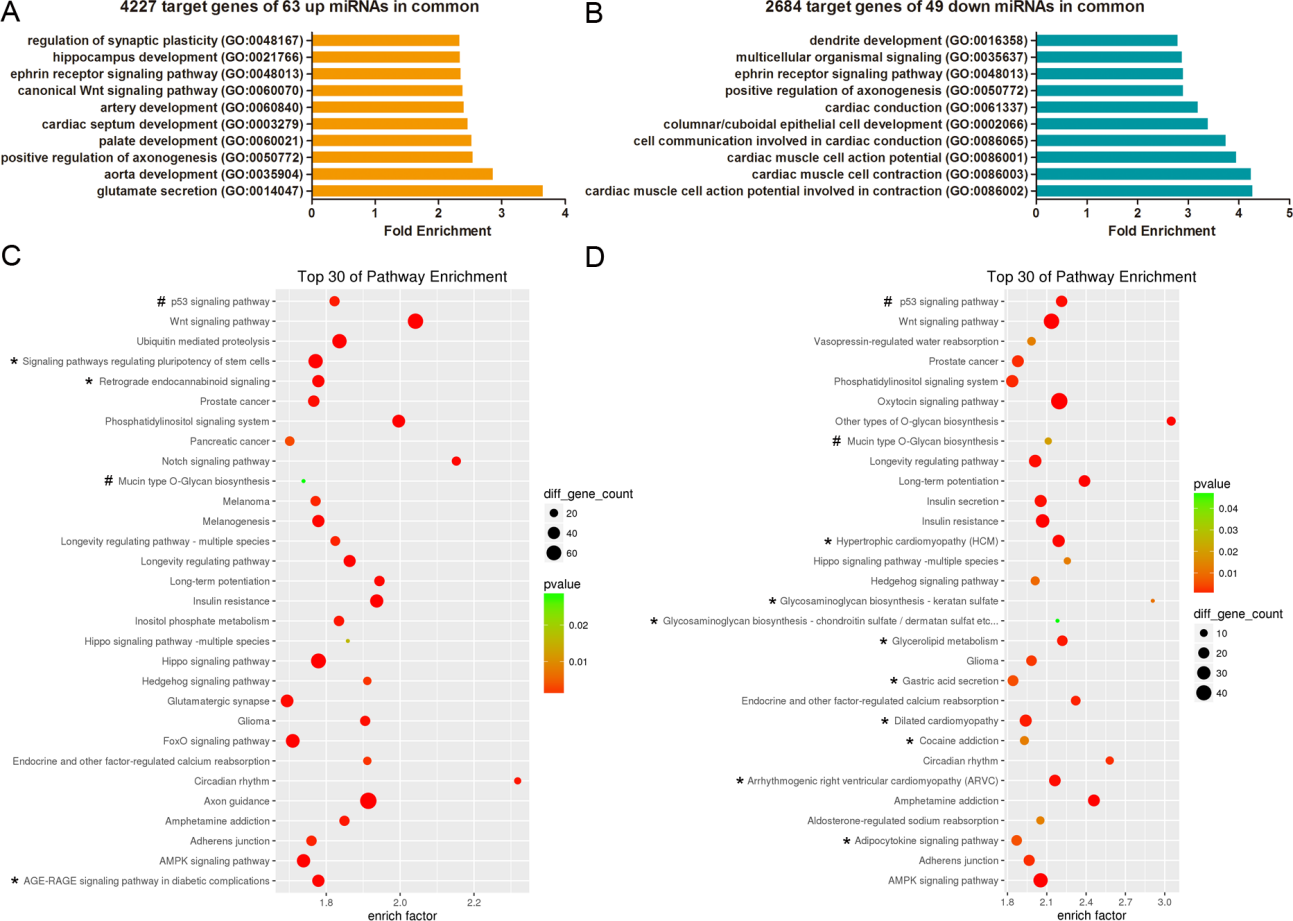
GO and KEGG pathway analyses show the associated function of the target genes of the miRNAs dysregulated in both CP and CLP (**A**) The top 10 enriched GO terms for the 4227 target genes of the 63 miRNAs upregulated in both CP and CLP. (**B**) The top 10 enriched GO terms for the 2684 target genes of the 49 miRNAs downregulated in both CP and CLP. (**C**) The top 30 enriched pathways for the 4227 target genes of the 63 miRNAs upregulated in both CP and CLP. (**D**) The top 30 enriched pathways for the 2684 target genes of the 49 miRNAs downregulated in both CP and CLP. (C-D) The enrichment *P* values were calculated using Fisher's exact test. The term/pathway on the vertical axis was drawn according to the first letter of the pathway name in descending order. The horizontal axis represents the enrichment factor, i.e., (the number of dysregulated genes in a pathway/the total number of dysregulated genes)/(the number of genes in a pathway in the database/the total number of genes in the database). The top 30 enriched pathways were selected according to the enrichment factor value. The selection standards were the number of genes in a pathway ≥ 4 and *p <* 0.05. The different colours from green to red represent the *p-value*. The different sizes of the round shapes represent the gene count number in a pathway. *indicates the pathway is similarly regulated in both CP and CLP. # means the pathway is regulated in both CP and CLP but the miRNA target genes may be upregulated or downregulated.

To explore whether overlapping biological processes were found in both CLP and CPO, we performed gene ontology (GO) and KEGG pathway analysis for the predicted targets of the differentially expressed miRNAs, including the 63 upregulated and 49 downregulated miRNAs in both CP and CLP, which produced 4227 and 2684 predictive target genes, respectively. The top ten enriched GO terms for the upregulated and downregulated are listed in Figure 3A and 3B, respectively. The analysis revealed that the potential target genes of the 63 miRNAs upregulated in both CP and CLP were associated with glutamate secretion, aorta or palate development, positive regulation of axonogenesis, cardiac septum development, artery development, the canonical Wnt signalling pathway, and the ephrin receptor signalling pathway (Figure 3A). Conversely, the potential target genes of the 49 miRNAs downregulated in both CP and CLP were associated with the generation of contraction-related action potentials in cardiac muscle cells, cardiac muscle cell contraction, columnar/cuboidal epithelial cell development, cardiac conduction, positive regulation of axonogenesis, and the ephrin receptor signalling pathway (Figure 3B). Pathway enrichment analysis revealed that the potential target genes of the miRNAs dysregulated in both CP and CLP were involved in p53 signalling, Wnt signalling, circadian rhythm, insulin resistance, and the AMPK signalling pathway (Figure 3C, 3D).

### Function analysis of the potential target genes of the miRNAs dysregulated in CP

**Figure 4:**
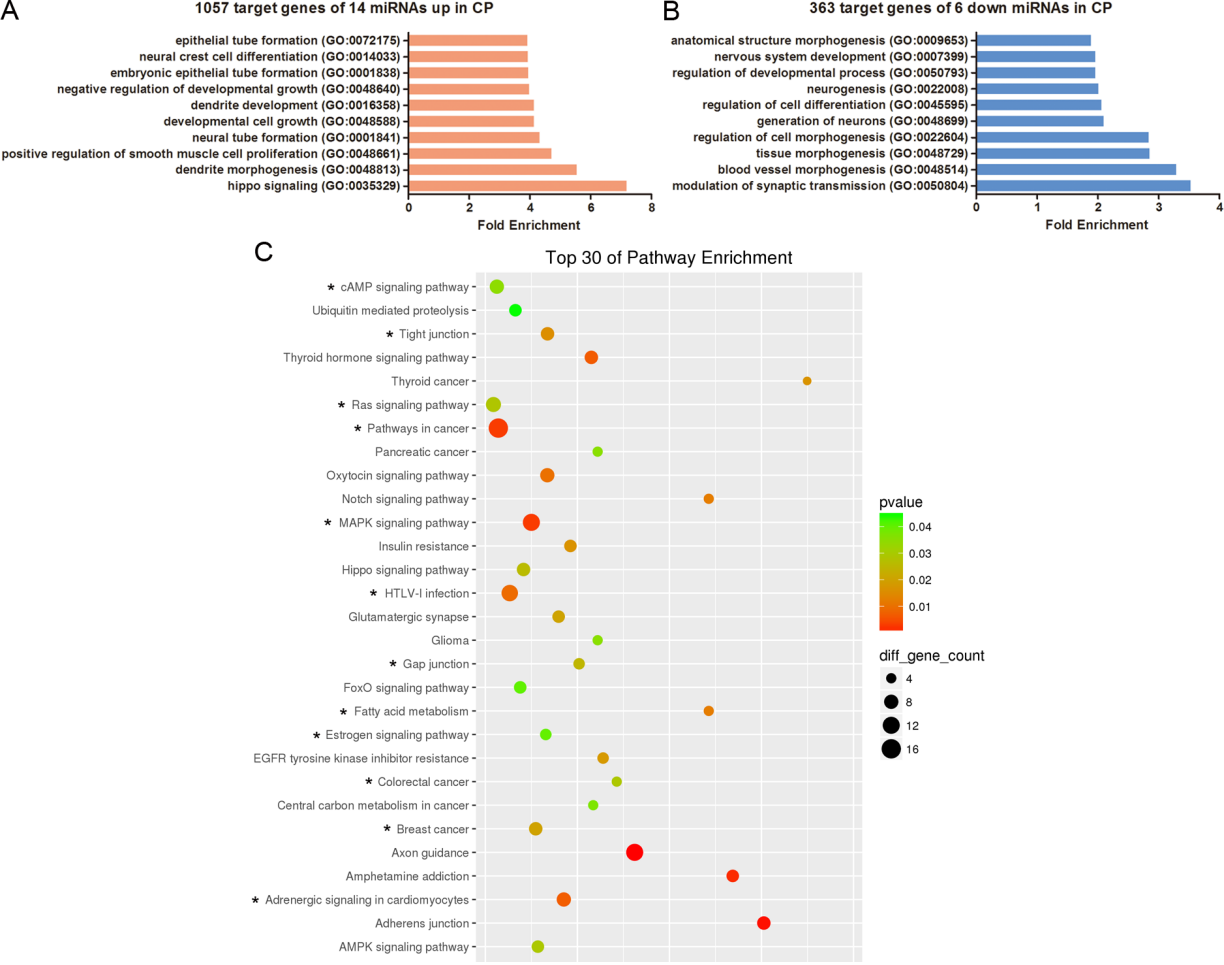
GO and KEGG pathway analyses show the associated function of the target genes of the miRNAs dysregulated in CP (**A**) The top 10 enriched GO terms for the 1057 target genes of the 14 miRNAs upregulated in CP. (**B**) The top 10 enriched GO terms for the 363 target genes of the 6 miRNAs downregulated in CP. (**C**) Top 30 enriched pathways for the 363 target genes of the 6 miRNAs downregulated in CP. The enrichment *P* values were calculated using Fisher's exact test. The term/pathway on the vertical axis was drawn according to the first letter of the pathway name in descending order. The horizontal axis represents the enrichment factor, i.e., (the number of dysregulated genes in a pathway/the total number of dysregulated gene)/(the number of genes in a pathway in the database/the total number of genes in the database). Top 30 enriched pathways were selected according to the enrichment factor value. The selection standards were the number of genes in a pathway ≥ 4 and *p <* 0.05. The different colours from green to red represent the *p value*. The different sizes of the round shapes represent the gene count number in a pathway. * indicates the pathway is similarly regulated in both CP and CLP.

To explore whether distinct biological processes regulated CLP and CPO, we performed gene ontology (GO) and KEGG analysis of the predicted targets of the differentially expressed miRNAs in CP, including 14 upregulated miRNAs producing 1057 predictive target genes and 6 downregulated miRNAs yielding 363 potential target genes. The top ten enriched GO terms are listed in Figure 4A and 4B, respectively. The analysis revealed that the potential target genes of the 14 miRNAs upregulated in CP were associated with hippo signalling, dendrite morphogenesis or development, positive regulation of smooth muscle cell proliferation, neural tube formation and developmental cell growth (Figure 4A). Conversely, the potential target genes of the 6 miRNAs downregulated in CP were associated with modulation of synaptic transmission, blood vessel or tissue morphogenesis, regulation of cell morphogenesis or cell differentiation and neurogenesis (Figure 4B). Surprisingly, 1057 of the predictive target genes of the 14 miRNAs upregulated in CP did not display KEGG enrichment results using the SBC analysis system. For the 363 potential target genes of the 6 miRNAs downregulateCP, the pathway enrichment results showed that those genes were mainly involved in thyroid cancer, the Notch signalling pathway, fatty acid metabolism, adherens junctions and amphetamine addiction (Figure 4C).

### Function analysis of the potential target genes of the miRNAs dysregulated in CLP

**Figure 5:**
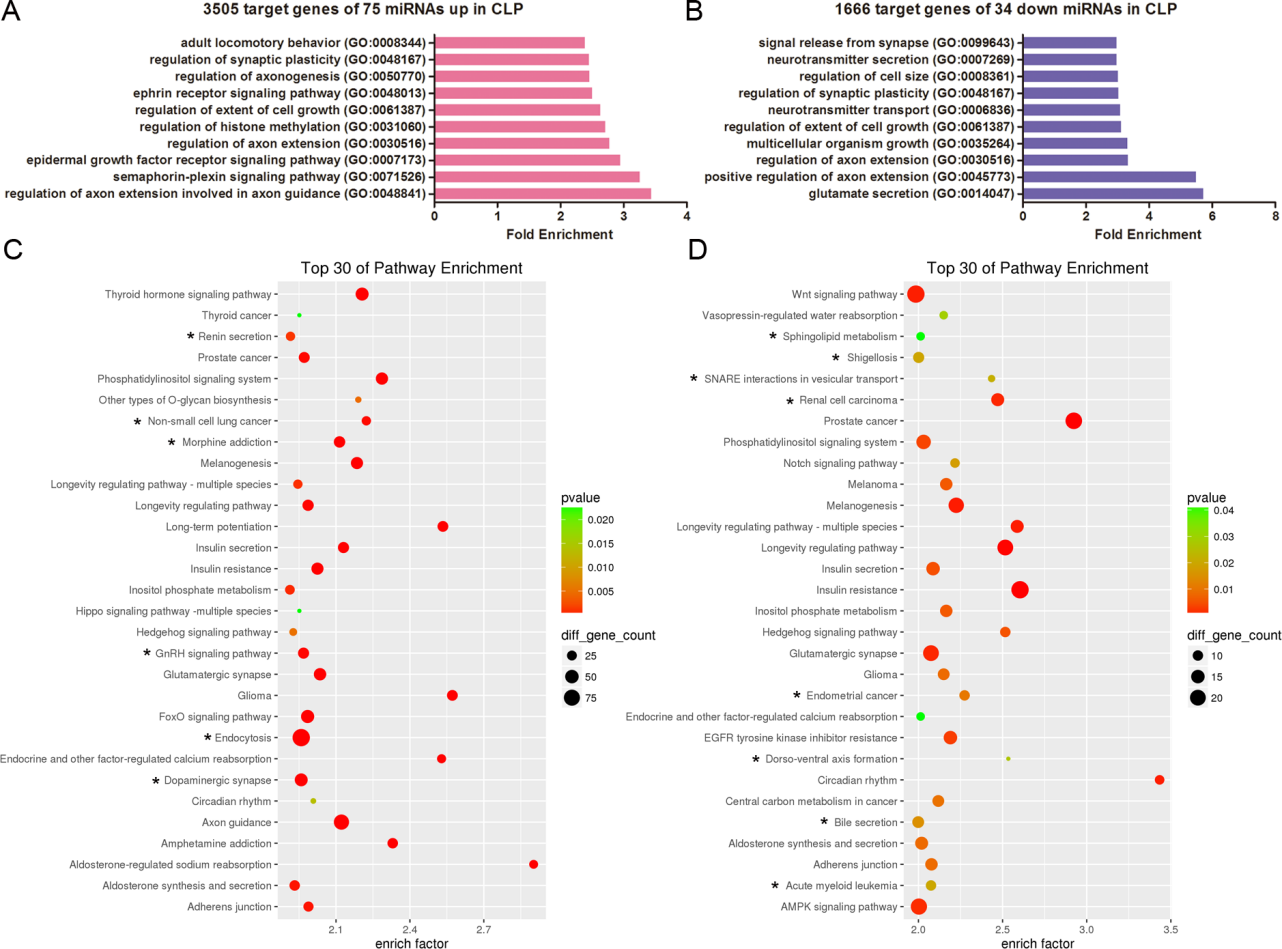
GO and KEGG pathway analyses show the associated function of the target genes of the miRNAs dysregulated in CLP (**A**) The top 10 enriched GO terms for the 3505 target genes of the 75 miRNAs upregulated in CLP. (**B**) The top 10 enriched GO terms for the 1666 target genes of the 34 miRNAs downregulated in CLP. (**C**) Top 30 enriched pathways for the 3505 target genes of the 75 miRNAs upregulated in CLP. (**D**) Top 30 enriched pathways for the 1666 target genes of the 34 miRNAs downregulated in CLP. (C–D) The enrichment *P* values were calculated using Fisher's exact test. The term/pathway on the vertical axis was drawn according to the first letter of the pathway name in descending order. The horizontal axis represents the enrichment factor, i.e., (the number of dysregulated genes in a pathway/the total number of dysregulated genes)/(the number of genes in a pathway in the database/the total number of genes in the database). The top 30 enriched pathways were selected according to the enrichment factor value. The selection standards were the number of genes in a pathway ≥ 4 and *p <* 0.05. The different colours from green to red represent the *p value*. The different sizes of the round shapes represent the gene count number in a pathway. * indicates the pathway is similarly regulated in both CLP and CP.

Additionally, we performed gene ontology (GO) and KEGG analysis of the predicted targets of the differentially expressed miRNAs in CLP, including 75 upregulated miRNAs yielding 3505 possible target genes and 34 downregulated miRNAs creating 1666 possible target genes. The top ten enriched GO terms are listed in Figure 5A and 5B, respectively. The analysis revealed that the potential target genes of the 75 miRNAs upregulated in CLP were associated with the regulation of axon extension during axon guidance, the semaphorin-plexin signalling pathway, the epidermal growth factor receptor signalling pathway, the regulation of axon extension, histone methylation and the extent of cell growth (Figure 5A). Conversely, the potential target genes of the 34 miRNAs downregulated in CLP were associated with glutamate secretion, the positive regulation of axon extension, multicellular organism growth, the extent of cell growth, neurotransmitter transport and the regulation of synaptic plasticity (Figure 5B). Pathway enrichment analysis indicated that the potential target genes of the miRNAs dysregulated in CLP were associated with specific pathways, including the thyroid hormone signalling pathway, the GnRH signalling pathway, the Hedgehog signalling pathway, and the longevity regulating pathway (Figure 5C, 5D).

## DISCUSSION

Plasma miRNAs are highly stabile under handling and storage conditions [12]. They play crucial roles in many diseases including cancer [13, 14] and respiratory diseases [15]. Nonsyndromic cleft palate and nonsyndromic cleft lip with cleft palate are two types of oral clefting that occur without other developmental syndromes. Research on the roles of differentially expressed plasma miRNAs in NSCP and NSCLP patients will improve our knowledge, diagnosis and management of the two diseases. This study, for the first time, has used microarray profiling to evaluate the differential expression of miRNAs in plasma samples from NSCP and NSCLP patients compared with healthy children. Six miRNAs, namely miR-340–5p, miR-877–5p, miR-3648, miR-1260a, miR-494–3p, and miR-1304–3p, were found to be differentially expressed in both the NSCP and NSCLP plasma samples.

Venn diagrams have been widely used to visualize the relationship between complex genetic data sets [16, 17]. Based on our miRNA microarray data, we found the intersection of differentially expressed miRNAs in CP and CLP. Using Bulge-Loop™ qRT-PCR analysis, we demonstrated that the upregulated and downregulated miRNAs were consistent with the results of the miRNA microarray assay. Therefore, we investigated the key genes and pathways associated with CP and/or CLP using bioinformatics analysis according to the microarray data. In mammals, miRNAs could regulate 30% of the protein-coding genes through posttranscriptional silencing; therefore, the dysregulation of miRNAs in NSCP or NSCLP patients could have a profound influence on various biological functions.

Based on the GO analysis, the predicted target genes of the upregulated and downregulated miRNAs in both CP and CLP mainly participated in glutamate secretion, aorta or palate development, cardiac muscle cell contraction, and the ephrin receptor signalling pathway. Examining the top 30 enriched pathways showed that the target genes were solely associated with several pathways, including the AGE-RAGE signalling pathway in diabetic complications, retrograde endocannabinoid signalling, signalling pathways regulating the pluripotency of stem cells, the adipocytokine signalling pathway, arrhythmogenic right ventricular cardiomyopathy (ARVC), cocaine addiction, dilated cardiomyopathy, gastric acid secretion, glycerolipid metabolism, glycosaminoglycan biosynthesis-chondroitin sulphate/dermatan sulphate, glycosaminoglycan biosynthesis-keratan sulphate, hypertrophic cardiomyopathy (HCM), the p53 signalling pathway and mucin type O-Glycan biosynthesis. Consistent with this prediction, cleft palate and cleft lip with cleft palate may be associated with a wide range of signalling molecules, including transforming growth factors (TGFs) [18], bone morphogenetic proteins (BMPs) [19], and fibroblast growth factors (FGFs) [20]. In addition, various developmental transcription factors belonging to the paired box (PAX) [21], distal-less homeobox (DLX) [22], msh homeobox (MSX) [23], and T-Box (TBX) gene families [24] are also involved in CP and CLP.

Recent studies suggest that isolated cleft palate only (CPO) has independent genetic causes and should be evaluated separately [25, 26]. Therefore, we analysed the predictive target genes of nonoverlapping miRNAs in CP and CLP using GO and KEGG pathway analysis. Interestingly, the top 10 enriched GO terms showed that Hippo signalling, dendrite morphogenesis, modulation of synaptic transmission and tissue morphogenesis were related to CP. Thirteen pathways of the top 30 enriched pathways were uniquely associated with CP including 2-oxocarboxylic acid metabolism, adrenergic signalling in cardiomyocytes, breast cancer, the cAMP signalling pathway, colorectal cancer, the oestrogen signalling pathway, fatty acid metabolism, gap junctions, HTLV-I infection, the MAPK signalling pathway, pathways in cancer, the ras signalling pathway, and tight junctions. In contrast, 14 pathways of the top 30 enriched pathways were only linked with CLP including dopaminergic synapses, endocytosis, the GnRH signalling pathway, morphine addiction, non-small cell lung cancer, renin secretion, acute myeloid leukaemia, bile secretion, dorso-ventral axis formation, endometrial cancer, renal cell carcinoma, shigellosis, SNARE interactions in vesicular transport, and Sphingolipid metabolism.

NSCP and NSCLP are developmental defects. The differentially expressed plasma miRNAs identified in the affected infants could be from their mother's blood. Further elucidation of the sources of the plasma miRNAs in human tissues and their roles in the pathogenesis of cleft palate or cleft lip with cleft palate, particularly in palate development or the regulation of cranial neural crest (CNC) cells that give rise to craniofacial structures, needs to be further explored. In addition, larger samples are needed to perform receiver operating characteristic (ROC) curve analysis to prove that some of the children plasma microRNAs are promising biomarkers.

Taken together, based on the Bulge-Loop™ qRT-PCR analysis and miRNA microarray assay, we uncovered a differential plasma miRNA expression profile in NSCP and NSCLP patients compared with healthy children. Using GO and KEGG pathway analysis, we found distinct and overlapping biological processes or signalling pathways involved in CP and CLP. Further studies should examine the mechanism of the potential target genes, which may provide new avenues for future clinical prevention and therapy.

## MATERIALS AND METHODS

### Sample collection

The study protocol was approved by the Institutional Review Board of Nanjing Medical University (2014–10–16). The plasma samples were collected according to previously described methods [11]. Nonsyndromic cleft palate (NSCP) and nonsyndromic cleft lip with cleft palate (NSCLP) patients who underwent surgery and healthy children (normal face but who underwent surgery for hydrocele) participated in this study with their parent's consent at the Department of Burns and Plastic Surgery, Nanjing Children's Hospital Affiliated to Nanjing Medical University in Nanjing, China. All NSCP, NSCLP and healthy children were between 2 and 12 months old. The patient information is listed in Table 4. Briefly, peripheral blood was collected into EDTAK2 tubes (regular type), and then immediately centrifuged at 1000 g for 15 min. The supernatant plasma was transferred to RNase-free tubes and centrifuged at 12000 g for 10 min to pellet any remaining cellular debris. Aliquots of the supernatant were transferred to fresh tubes and immediately stored at –80**°**C.

**Table 4:** Patient characteristics

Group	Age (m, months; d, days)	Gender	Sample use
Control	9m12d	female	Array
Control	3m30d	male	Array
Control	10m	male	Array
Control	6m	female	qRT-PCR
Control	9m	female	qRT-PCR
Control	12m	female	qRT-PCR
Control	3m	male	qRT-PCR
Control	9m	male	qRT-PCR
Control	3m	male	qRT-PCR
Control	6m	male	qRT-PCR
Control	5m	male	qRT-PCR
CP	3m	female	Array
CP	6m	female	Array
CP	5m	male	Array
CP	10m7d	female	qRT-PCR
CP	12m	female	qRT-PCR
CP	3m	female	qRT-PCR
CP	8m28d	female	qRT-PCR
CP	9m20d	female	qRT-PCR
CP	10m9d	female	qRT-PCR
CP	11m13d	female	qRT-PCR
CP	8m	female	qRT-PCR
CP	12m	female	qRT-PCR
CP	12m	female	qRT-PCR
CLP	7m30d	male	Array
CLP	4m	male	Array
CLP	4m21d	male	Array
CLP	2m22d	female	qRT-PCR
CLP	4m17d	female	qRT-PCR
CLP	12m	female	qRT-PCR
CLP	10m25d	female	qRT-PCR
CLP	12m	female	qRT-PCR
CLP	10m24d	female	qRT-PCR
CLP	5m	female	qRT-PCR
CLP	7m	female	qRT-PCR
CLP	12m	female	qRT-PCR
CLP	11m	female	qRT-PCR
CLP	3m21d	female	qRT-PCR
CLP	5m30d	female	qRT-PCR
CLP	4m12d	male	qRT-PCR
CLP	3m12d	male	qRT-PCR
CLP	4m23d	male	qRT-PCR
CLP	3m20d	male	qRT-PCR
CLP	2m10d	male	qRT-PCR
CLP	2m	male	qRT-PCR
CLP	9m29d	male	qRT-PCR
CLP	12m	male	qRT-PCR
CLP	12m	male	qRT-PCR
CLP	11m3d	male	qRT-PCR
CLP	3m	male	qRT-PCR
CLP	6m	male	qRT-PCR
CLP	9m10d	male	qRT-PCR
CLP	12m	male	qRT-PCR
CLP	11m	male	qRT-PCR
CLP	4m21d	male	qRT-PCR
CLP	3m30d	male	qRT-PCR
CLP	11m12d	male	qRT-PCR

CP, cleft palate; CLP, cleft lip with palate.

### Total RNA isolation from human plasma samples

Total RNA was isolated from 400 μl of human plasma samples using the mirVana PARIS kit (Ambion, Carlsbad, CA, USA) according to the manufacturer's instructions. After infusing an equal volume of 2x denaturing solution to the plasma samples to inactivate RNases, the denatured samples were mixed with Synthetic Caenorhabditis elegans miRNA cel-miR-39 (GenePharma, Shanghai, China), to normalize the variation in RNA isolation from the different samples. RNA was eluted using 100 μl of elution solution.

### Mature miRNA microarray analysis

Nine samples, which included three NSCP plasma samples (mixed as the CP group), three NSCLP plasma samples (mixed as the CLP group) and three normal plasma samples (mixed as the Control group), were analysed using Agilent human miRNA microarray chips (8*60K) v21.0 (ShanghaiBio Corporation, Shanghai, China). The raw data were normalized using the quantile algorithm in GeneSpring Software 12.6 (Agilent Technologies, Santa Clara, CA, USA). The differentially expressed miRNAs that showed a two-fold or greater change were screened. Venn diagrams were draw online (http://bioinformatics.psb.ugent.be/webtools/Venn/).

### Validation of the microarray data using Bulge-Loop™ miRNA qRT-PCR

To confirm the microarray data, six selected miRNAs with two-fold or greater changes were further validated in samples from an additional 16 CP, 33 CLP and 8 healthy children using Bulge-Loop™ qRT-PCR according to the manufacturer's protocol (RIBOBIO, Guangzhou, China) with SYBR green on an Applied Biosystems ViiA™ 7 Dx (Life Technologies, USA). The expression levels of the miRNAs were normalized to C. elegans control miRNA cel-39 using the 2^(–△△Ct)^ method [27].

### Statistical analysis

The validation results from the qRT-PCR analysis are displayed as the mean ± SD. Statistical significance was assessed using the Mann-Whitney *U* test in Graphpad Prism 6 (Graphpad software, CA, USA). A *P <* 0.05 was considered statistically significant.

### GO and pathway enrichment analyses

The differentially expressed miRNAs were further analysed for predicted gene targets simultaneously using at least two of the following five databases: TARGETMINER, miRDB, microRNA, TarBase, and RNA22 through the ShanghaiBio Corporation (SBC) analysis system (http://sas.ebioservice.com). GO enrichment analyses were performed online (http://geneontology.org/). KEGG pathway enrichment analyses of the predicted targets of the differentially expressed miRNAs were performed according to previously described methods [11]. KEGG pathway enrichment analyses were performed by the ShanghaiBio Corporation (SBC) analysis system, which uses clusterProfiler data from R/bioconductor software (http://www.r-project.org and http://www.bioconductor.org/) with public databases that include NCBI Entrez Gene (http://www.ncbi.nlm.nih.gov/gene), GO (http://www.geneontology.org), KEGG (http://www.genome.jp/kegg), and Biocarta (http://www.biocarta.com). The enrichment *P*-values of both the GO and pathway enrichment analyses were calculated using the Fisher's exact test [28], which was corrected using enrichment *q*-values (the false discovery rate) that were calculated using John Storey's method [29].

## References

[1] Rahimov F, Jugessur A, Murray JC (2012). Genetics of nonsyndromic orofacial clefts. Cleft Palate Craniofac J.

[2] Tolarova MM, Cervenka J (1998). Classification and birth prevalence of orofacial clefts. Am J Med Genet.

[3] Burg ML, Chai Y, Yao CA, Magee W, Figueiredo JC (2016). Epidemiology, Etiology, and Treatment of Isolated Cleft Palate. Front Physiol.

[4] Sivertsen A, Wilcox AJ, Skjaerven R, Vindenes HA, Abyholm F, Harville E, Lie RT (2008). Familial risk of oral clefts by morphological type and severity: population based cohort study of first degree relatives. BMJ.

[5] Leslie EJ, Marazita ML (2013). Genetics of cleft lip and cleft palate. Am J Med Genet C Semin Med Genet.

[6] Peng HH, Chang NC, Chen KT, Lu JJ, Chang PY, Chang SC, Wu-Chou YH, Chou YT, Phang W, Cheng PJ (2016). Nonsynonymous variants in MYH9 and ABCA4 are the most frequent risk loci associated with nonsyndromic orofacial cleft in Taiwanese population. BMC Med Genet.

[7] Kondo S, Schutte BC, Richardson RJ, Bjork BC, Knight AS, Watanabe Y, Howard E, de Lima RL, Daack-Hirsch S, Sander A, McDonald-McGinn DM, Zackai EH, Lammer EJ (2002). Mutations in IRF6 cause Van der Woude and popliteal pterygium syndromes. Nat Genet.

[8] Radhakrishna U (2012). Small players with a big role: MicroRNAs in pathophysiology of cleft lip and palate. Indian J Hum Genet.

[9] Wang J, Bai Y, Li H, Greene SB, Klysik E, Yu W, Schwartz RJ, Williams TJ, Martin JF (2013). MicroRNA-17–92, a direct Ap-2alpha transcriptional target, modulates T-box factor activity in orofacial clefting. PLoS Genet.

[10] Warner DR, Mukhopadhyay P, Brock G, Webb CL, Michele Pisano M, Greene RM (2014). MicroRNA expression profiling of the developing murine upper lip. Dev Growth Differ.

[11] Zou J, Li J, Li J, Ji C, Li Q, Guo X (2016). Expression profile of plasma microRNAs in nonsyndromic cleft lip and their clinical significance as biomarkers. Biomed Pharmacother.

[12] Balzano F, Deiana M, Dei Giudici S, Oggian A, Baralla A, Pasella S, Mannu A, Pescatori M, Porcu B, Fanciulli G, Zinellu A, Carru C, Deiana L (2015). miRNA Stability in Frozen Plasma Samples. Molecules.

[13] Li C, Li JF, Cai Q, Qiu QQ, Yan M, Liu BY, Zhu ZG (2013). miRNA-199a-3p in plasma as a potential diagnostic biomarker for gastric cancer. Ann Surg Oncol.

[14] Torres A, Torres K, Pesci A, Ceccaroni M, Paszkowski T, Cassandrini P, Zamboni G, Maciejewski R (2013). Diagnostic and prognostic significance of miRNA signatures in tissues and plasma of endometrioid endometrial carcinoma patients. Int J Cancer.

[15] Molina-Pinelo S, Suarez R, Pastor MD, Nogal A, Marquez-Martin E, Martin-Juan J, Carnero A, Paz-Ares L (2012). Association between the miRNA signatures in plasma and bronchoalveolar fluid in respiratory pathologies. Dis Markers.

[16] Yang JJ, Kwon H, Lee JM (2016). Complementary Characteristics of Correlation Patterns in Morphometric Correlation Networks of Cortical Thickness, Surface Area, and Gray Matter Volume. Sci Rep.

[17] Kestler HA, Muller A, Gress TM, Buchholz M (2005). Generalized Venn diagrams: a new method of visualizing complex genetic set relations. Bioinformatics.

[18] Lane J, Yumoto K, Azhar M, Ninomiya-Tsuji J, Inagaki M, Hu Y, Deng CX, Kim J, Mishina Y, Kaartinen V (2015). Tak1, Smad4 and Trim33 redundantly mediate TGF-beta3 signaling during palate development. Dev Biol.

[19] Cela P, Hampl M, Fu KK, Kunova Bosakova M, Krejci P, Richman JM, Buchtova M (2016). MORN5 Expression during Craniofacial Development and Its Interaction with the BMP and TGFbeta Pathways. Front Physiol.

[20] Wu W, Gu S, Sun C, He W, Xie X, Li X, Ye W, Qin C, Chen Y, Xiao J, Liu C (2015). Altered FGF Signaling Pathways Impair Cell Proliferation and Elevation of Palate Shelves. PLoS One.

[21] Sull JW, Liang KY, Hetmanski JB, Fallin MD, Ingersoll RG, Park J, Wu-Chou YH, Chen PK, Chong SS, Cheah F, Yeow V, Park BY, Jee SH (2009). Maternal transmission effects of the PAX genes among cleft case-parent trios from four populations. Eur J Hum Genet.

[22] Wu D, Mandal S, Choi A, Anderson A, Prochazkova M, Perry H, Gil-Da-Silva-Lopes VL, Lao R, Wan E, Tang PL, Kwok PY, Klein O, Zhuan B (2015). DLX4 is associated with orofacial clefting and abnormal jaw development. Hum Mol Genet.

[23] Singh N, Gupta M, Trivedi CM, Singh MK, Li L, Epstein JA (2013). Murine craniofacial development requires Hdac3-mediated repression of Msx gene expression. Dev Biol.

[24] Funato N, Nakamura M, Richardson JA, Srivastava D, Yanagisawa H (2012). Tbx1 regulates oral epithelial adhesion and palatal development. Hum Mol Genet.

[25] Rahimov F, Marazita ML, Visel A, Cooper ME, Hitchler MJ, Rubini M, Domann FE, Govil M, Christensen K, Bille C, Melbye M, Jugessur A, Lie RT (2008). Disruption of an AP-2alpha binding site in an IRF6 enhancer is associated with cleft lip. Nat Genet.

[26] Jugessur A, Shi M, Gjessing HK, Lie RT, Wilcox AJ, Weinberg CR, Christensen K, Boyles AL, Daack-Hirsch S, Nguyen TT, Christiansen L, Lidral AC, Murray JC (2011). Fetal genetic risk of isolated cleft lip only versus isolated cleft lip and palate: a subphenotype analysis using two population-based studies of orofacial clefts in Scandinavia. Birth Defects Res A Clin Mol Teratol.

[27] Livak KJ, Schmittgen TD (2001). Analysis of relative gene expression data using real-time quantitative PCR and the 2(-Delta Delta. C(T)) Method. Methods.

[28] Fisher RA (1922). On the interpretation of x(2) from contingency tables, and the calculation of P. Journal of the Royal Statistical Society.

[29] Storey JD, Taylor JE, Siegmund D (2004). Strong control, conservative point estimation and simultaneous conservative consistency of false discovery rates: a unified approach. Journal of the Royal Statistical Society.

